# A decade of liver transplantation in Mongolia: Economic insights and cost analysis

**DOI:** 10.1186/s13561-024-00528-0

**Published:** 2024-07-19

**Authors:** Amarjargal Tsengel, Sergelen Orgoi, Otgonbayar Damdinbazar, Bat-Ireedui Badarch, Urnultsaikhan Ganbold, Batsaikhan Batsuuri, Yerkyebulan Mukhtar, Batsaikhan Bat-Erdene, Liu Lei, Tserenbat Bazarsad, Undarmaa Zandanbazar, Gantugs Yundendorj

**Affiliations:** 1 First Central Hospital of Mongolia, Ulaanbaatar, Mongolia; 2 First Central Hospital of Mongolia, Organ Transplantation Center, Ulaanbaatar, Mongolia; 3 Division for Science and Technology, Mongolian National University of Medical Sciences, Ulaanbaatar, Mongolia; 4 Diagnostic Imaging Center, First Central Hospital of Mongolia, Ulaanbaatar, Mongolia; 5 Department of Epidemiology and Biostatistics, School of Public Health, Mongolian National University of Medical Sciences, Ulaanbaatar, Mongolia; 6 Department of Surgery, Mongolian National University of Medical Sciences, Ulaanbaatar, Mongolia; 7 Guangdong provincial hospital, Southern Medical University, Baiyun, China; 8 Department of Health Policy, School of Public Health, Mongolian National University of Medical Sciences, Ulaanbaatar, Mongolia

**Keywords:** Liver transplant surgery, Treatment costs, Disease severity, Mongolia

## Abstract

**Background:**

Mongolia introduced liver transplantation 10 years ago, becoming the 46th country globally to successfully perform this procedure. However, the cost of liver transplantation treatment remains expensive in Mongolia, a lower-middle-income country. Thus, the need to calculate the cost of liver transplants, a highly-valued treatment, forms the basis for this study.

**Methods:**

This study employed a retrospective research design with secondary data. The primary dataset comprised 143 cases of liver transplantation performed at the First Central Hospital of Mongolia between 2011 and 2021.

**Results:**

The average cost of a liver transplant in Mongolia is $39,589 ± 10,308, with 79.6% being direct costs and 20.4% indirect costs. Of the direct costs, 71% were attributed to drugs, medical equipment, and supplies, while 8.6% accounted for salaries. In terms of the Model of End-Stage Liver Disease (MELD) scores, treatment costs were $39,205 ± 10,786 for patients with MELD ≤ 14 points, $40,296 ± 1,517 for patients with MELD 15–20 points, $39,352 ± 8,718 for patients with MELD 21–27 points, and $39,812 ± 9,954 for patients with MELD ≤ 28 points, with no statistically significant difference (*P* = 0.953). However, when calculated according to the Child-Turcotte-Pugh (CTP) score classification, treatment cost for CTP-A patients was $35,970 ± 6,879, for CTP-B patients $41,951 ± 12,195, and for CTP-C patients $37,396 ± 6,701, which was statistically significant (Р=0.015).

**Conclusion:**

The average cost of liver transplantation treatment in Mongolia was $39,589. Despite medical facilities’ capacity to treat up to 50 patients annually, the waiting list exceeds 300 individuals, highlighting significant unmet healthcare needs.

## Introduction

Mongolia, a developing country in Central Asia, had an estimated total population of 3.4 million in 2022, ranking 133rd globally [1]. The country faces a high prevalence of Hepatitis B and C infections, while hepatocellular carcinoma (HCC) is the predominant form of cancer, having a prevalence rate of 54.1 cases per 100,000 population [2]. According to the World Health Organization (WHO), over 19.2 million people were diagnosed with cancer in 2020, with 9.9 million cancer-related deaths worldwide [3]. Mongolia has one of the highest cancer mortality rates globally, with liver cancer accounting for 39.1% of all cancer cases, equivalent to 7.3 cases per 10,000 population—an increase of 0.2 cases from the previous year [4, 5].

A 2019 survey revealed that 19.4% of the adult population in Mongolia tested positive for either hepatitis B virus (HBV) or hepatitis C virus (HCV) infection. The estimated prevalence of HBV infection in the country ranges from 9 to 11.8%, while that of HCV ranges from 8.5 to 11.0%. In contrast, high-income countries, such as the United States (US), have much lower incidence rates: 0.3–0.7% for HBV and 1–1.6% for HCV [6].

Over the past decade, Mongolia recorded an average of 16,900 deaths annually due to cardiovascular diseases (33.3%), cancers (24.5%), and injuries and external causes (16.5%). In 2022, the mortality rate for cancer was estimated at approximately 23.7% of total deaths. This estimation is based on a mortality rate of 15.2 per 10,000 men and 10.4 per 10,000 women. Liver disease remains the leading cause of death from liver cancer in Mongolia, with a mortality rate eight times higher than the global average [4].

Liver transplantation (LT) is a potentially life-saving and life-prolonging intervention, widely recognized as the standard treatment for patients with end-stage liver disease or acute liver failure [7]. The world’s first successful liver transplant took place in 1968, and this treatment has made significant progress in all developed countries. Mongolia introduced LT in 2011 at the First Central Hospital of Mongolia (FCHM), becoming the 46th country to perform liver transplant surgery [8].

Initially, patients in Mongolia paid 100% of LT treatment costs. Since 2018, the state budget and health insurance fund have covered these costs, with the FCHM receiving financing for 50 cases annually based on its capacity. In 2021, Mongolia reported a total of 181 transplant cases, with 143 cases specifically involving liver transplants performed at the FCHM [9]. Since 2004, 305 Mongolian patients have undergone LT abroad, with 60% (182) in India and 28% (87) in Korea [10].

Western countries support organ transplantation from deceased donor liver transplant (DDLT), whereas living donor liver transplantation (LDLT) is more common in Asian countries [11]. In Mongolia, the Donor law was first enacted in 2000 and amended in 2018 to revise the legal framework for protecting human health; saving lives through voluntary and unpaid donation and transplantation of tissues, and organs, and ensuring blood safety [12].

Recent global economic analysis frequently examined the association between the Model of End-Stage Liver Disease (MELD) score and procedure-related expenses, emphasizing the importance of prioritizing transplant criteria based on the severity of liver disease [13]. The Activity-Based Costing (ABC) method, widely used for over 30 years [14, 15], has primarily been applied in healthcare and manufacturing sectors for research purposes. Nobly in an unstable economic environment or during inflation, using the ABC method provides detailed and valuable cost information, which helps in making informed decisions [16].

It has now been a decade since the first liver transplant was performed at the FCHM in 2011. However, as research studies designed to examine data over this 10-year period are almost nonexistent, this premise forms the rationale for our study.

## Materials and methods

### Study population

This study employed a retrospective study design and used secondary data from patient records. Costing estimation was performed using the ABC methodology. Using statistical data collected between 2011 and 2021, a total of 143 patient records were obtained from the FCHM database. All cases registered at the hospital during this period were evaluated, and data from medical records and registration forms were analyzed.

The study data included the following variables: age, gender, disease diagnosis, cause of disease, liver disease MELD score [17], Child-Turcotte Pugh (CTP) score [18], length of hospitalization, duration of surgery, records of repeated surgery, treatment-related complications, amount of medications and medical appliances, blood products during treatment, and the number of diagnostic and analytic tests. The National Liver Transplantation Team selected patients eligible for liver transplantation in accordance with the Liver Transplantation Guidelines formally approved by Order No. A/19 of the Minister of Health of Mongolia, dated 26 January 2018 [7]. Conversely, no specific exclusion criteria were employed as the study included the entire sample of liver transplantation cases at the FCHM.

### Costing analysis-estimation

The ABC method was used to calculate the cost of LT [19]. The unit cost of services provided by the hospital served as the cost center utilized for determining treatment costs. The top-down cost distribution method was used to calculate indirect costs, while the ABC cost distribution method was used to calculate direct costs from the hospital registration forms. For direct costs, the number of diagnoses and tests were recorded along with the cost of laboratory reagents being allocated per unit of service. In addition, the cost of medication, medical appliances, and blood products used by the patient was obtained directly from the patient’s medical history. Furthermore, the average salaries of both physicians and nurses during the patient’s treatment and hospital stay were used to calculate salary costs. Other costs were calculated as the sum of the costs incurred by each department involved in patient care. When calculating the cost of transplantation surgery using the ABC approach, we categorized costs into the following groups: wage costs, cost of drugs and medical supplies, laboratory costs (kits, reagents), and fixed costs.

### Variables

The variables included disease diagnosis, causes of the disease, severity of liver disease indicated by the MELD score, CTP score during treatment, length of hospital stay, duration of surgery, notes on repeated surgeries, treatment-related complications, and other variables involved in the treatment process, such as medications, medical equipment, volume of blood products administered, and number of diagnostic and analysis tests conducted.

### Statistical analysis

The data were analyzed using SPSS version 25.0. A t-test or one-way ANOVA test was performed to calculate the mean difference between groups. Pearson’s chi-squared test was used to calculate the percentage difference between the groups. A p-value of less than 0.05 was considered to indicate statistical significance.

## Results

Of the cases included in the study, 2 (1.3%) were aged 0–9 years, 5 (3.5%) were aged 10–19 years, 5 (3.5%) were aged 20–29 years, 30 (21%) were aged 30–39 years, 51 (35.7%) were aged 40–49 years, 36 (26%) were aged 50–59 years, and 14 (9%) were aged 60–69 years. Of these, 75 (52.4%) were men, and 68 (47.6%) were women.

The number of LT surgeries performed annually has been increasing. In 2011, 3 (2.1%) surgeries were performed, but this number has been steadily rising since 2018. By 2021, 31 LT surgeries were performed, accounting for 21.7% of the total. Of the 143 patients who underwent LT, 125 (87.4%) were diagnosed with HBV, 107 (74.8%) with HDV (Hepatitis D virus), and 29 (20.3%) with HCC. Regarding complications, 62 (43.4%) patients had a MELD score ≤ 14, 52 (36.4%) had a score of 15–20, 21 (14.7%) had a score of 21–27, and 8 (5.6%) had a score ≥ 28. According to the CTP score, 16 (11.2%) patients had a CTP-A score, 76 (53.1%) had a CTP-B score, and 51 (35.7%) had a CTP-C score. The MELD (*p* = 0.075) and CTP (*p* = 0.292) scores were not significantly associated with post-operative mortality. Notably, no statistical significance (*p* = 0.273) was observed for the two cases (1.4%) that required repeat surgery. Thus, there were 21 cases of deaths after LT over a 10-year period, regardless of the patient’s complication score, age, sex, or re-operation. In this study, there were 9 (6%) deceased donors and 134 (94%) living donors (Table 1).

**Table 1:** Demographic and clinical characteristics of liver transplant recipients

Variables	Total†	Status‡		*P* value
		Alive	Death	
	*n* (%)	*n* (%)	*n* (%)	
**Age (years)**				0.272§
0–9	2(1.3)	2 (100)	0(0)	
10–19	5 (3.5)	4 (80)	1 (1)	
20–29	5 (3.5)	2 (40)	3 (60)	
30–39	30 (2)	24 (80)	6 (1)	
40–49	51 (35.7)	47 (92.2)	4 (7.8)	
50–59	36 (3)	31 (86)	5 (4)	
60–69	14 (5)	12 (86)	2 (4)	
**Sex**				0.641¶
Female	68 (47.6)	59 (86.8)	9 (13.2)	
Male	75 (52.4)	63 (84)	12 (6)	
**Number of transplants per year**				0.846§
2011	3 (2.1)	3 (100)	0 (0)	
2012	4 (2.8)	3 (75)	1 (7)	
2013	4 (2.8)	4 (100)	0 (0)	
2014	5 (3.5)	3 (60)	2 (40)	
2015	6 (4.2)	6 (100)	0 (0)	
2016	9 (6.3)	7 (77.8)	2 (22.2)	
2017	9 (6.3)	7 (77.8)	2 (22.2)	
2018	23 (16.1)	20 (87)	3 (8)	
2019	26 (18.2)	22 (84.6)	4 (15.4)	
2020	23 (16.1)	21 (91.3)	2 (8.7)	
2021	31 (21.7)	26 (83.9)	5 (16.1)	
**Aetiology**				
**HCV**				1ɫ
Yes	14 (9.8)	12 (85.7)	2 (14.3)	
No	129 (90.2)	110 (85.3)	19 (14.7)	
**HBV**				0.730ɫ
Yes	125 (87.4)	107 (85.6)	18 (14.4)	
No	18 (12.6)	15 (83.3)	3 (16.7)	
**HDV**				0.698¶
Yes	107 (74.8)	92 (86)	15 (4)	
No	36 (25.2)	30 (83.3)	6 (16.7)	
**Biliary atresia**				1ɫ
Yes	3 (2.1)	3 (100)	0 (0)	
No	140 (97.9)	119 (85)	21 (9)	
**HCC**				0.568ɫ
Yes	29 (20.3)	26 (89.7)	3 (10.3)	
No	114 (79.7)	96 (84.2)	18 (15.8)	
**Meld score group**				0.475§
≤ 14	62 (43.4)	53 (85.5)	9 (14.5)	
15–20	52 (36.4)	46 (88.5)	6 (11.5)	
21–27	21 (14.7)	17 (81)	4 (10)	
≥ 28	8 (5.6)	6 (75)	2 (7)	
**CTP score**				0.292§
CTP-A	16 (11.2)	15 (93.8)	1 (6.3)	
CTP-B	76 (53.1)	65 (85.5)	11 (14.5)	
CTP-C	51 (35.7)	42 (82.4)	9 (17.6)	
**Retransplantation**				0.273ɫ
No	141 (98.6)	121 (85.8)	20 (14.2)	
Yes	2 (1.4)	1 (50)	1 (50)	
Donor				0.484ɫ
Living donor	134 (94)	115 (87)	19 (8)	
Deceased donor	9 (11)	7 (78)	2 (12)	
**Total**	**143 (100)**	**122 (85.3)**	**21 (14.7)**	

CTP - Child-Turcotte-Pugh; MELD - Model for End-stage Liver Disease; ‡ - Status: the share of this was calculated according to the line; § - Pearson’s chi-square test for trend; ¶ - Pearson’s chi-square test; ɫ - Fisher’s exact test

**Fig. 1:**
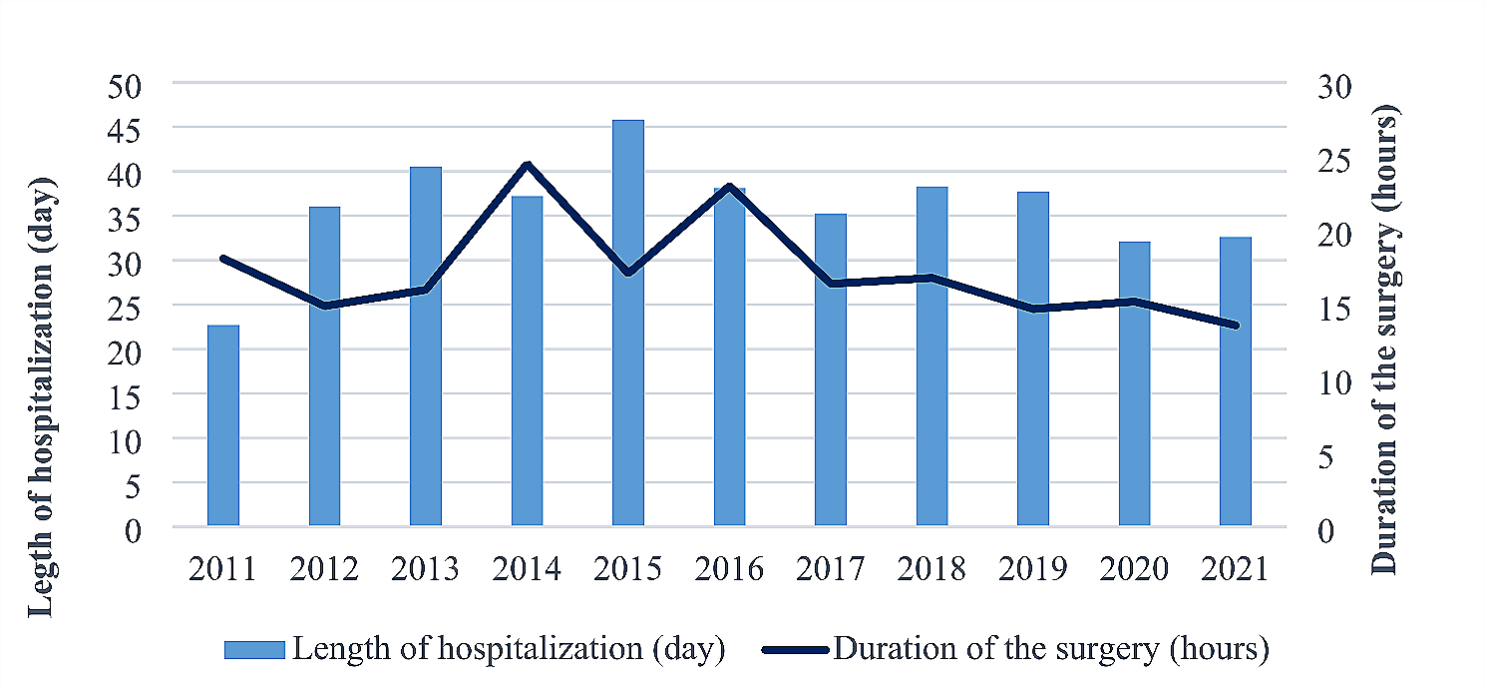
Length of hospital stay and duration of surgery

In 2011, the average length of hospital stay for patients was 22.7 ± 5.51 days, and in 2021 it was 32.6 ± 9.26 days. The duration of surgery decreased from 18.1 ± 6.2 h in 2011 to 13.6 ± 1.64 h in 2021. The longest recorded surgery duration was in 2014, lasting 24.5 ± 10.21 h, varying based on the severity of the patient’s condition (Fig. 1).

By December 2023, an assessment was conducted based on monitoring patients who had undergone LT surgery. On average, the survival period after LT is 108.3 ± 6.3 months for males and 129.7 ± 5.8 months for females. For patients with MELD scores ≤ 14, the survival period was 126.7 ± 6.8 months; for those with 15–20, it was 130.9 ± 6.3 months; for those with 21–27, it was 92.2 ± 8.9 months; and for those with MELD scores ≥ 28, it was 76.3 ± 14.1 months. When assessed by CTP scores, the survival period was as follows: CTP-A 97 ± 6 months, CTP-B 127.6 ± 5.8 months, and CTP-C 83.7 ± 4.8 months. For patients who had undergone retransplantation, the survival period was 51.4 ± 31.9 days. Overall, the survival period after LT was 126.9 ± 4.4 days (Table 2).

**Table 2:** Survival period after liver transplantation

Variables	Duration ^a^ ± SD (by month)	95% CI	Р value
**Gender**			0.573
Female	129.7 ± 5.8	118.2–141.1	
Male	108.3 ± 6.3	96– 120.6	
**Meld score**			0.683
≤ 14	126.7 ± 6.8	113.5–140	
15–20	130.9 ± 6.3	118.5–143.2	
21–27	92.2 ± 8.9	74.7–109.7	
≥ 28	76.3 ± 14.1	48.6–104	
**CTP score**			0.558
CTP-A	97 ± 6	85.2–108.8	
CTP-B	127.6 ± 5.8	116.1–139	
CTP-C	83.7 ± 4.8	74.3–93.1	
**Retransplantation**			0.129
No	127.6 ± 4.4	119–136.2	
Yes	51.4 ± 31.9	40.1–113.8	
**Total**		**126.9 ± 4.4**	**118.3**–**135.5**

If a patient is alive, survival time evaluated up to the date of the last examination

*P* value for the log rank test

The mean cost of LT was $39,657 ± 10,274, with the highest costs for post-operative drugs and medical supplies being $13,802 ± 6,116 (34.8%) for the recipient, $987 ± 663 (2.5%) for the donor, and the costs for surgery drugs and medical supplies being $4,076 ± 2,085 (10.3%) for the recipient and $4,699 ± 1,736 (11.9%) for the donor. The cost of drugs and medical supplies during anesthesia was $4,720 ± 1,633 (11.9%) for the recipient and $457 ± 628 (1.2%) for the donor (Table 3).

**Table 3:** Cost estimation of liver transplantation by expenses

Types of expenses		Cost /US dollar/		
		Mean	SD	%
Anesthetic drugs and equipment	Recipient	4,720	1,633	11.9
	Donor	457	628	1.2
Medication and instruments during surgery	Recipient	4,076	2,085	10.3
	Donor	4,699	1,736	11.9
Drugs and equipment after surgery	Recipient	13,802	6,116	34.8
	Donor	987	663	2.5
Blood and blood products		909	1,269	2.3
Diagnosis - Analysis	Recipient	2,950	1,218	7.4
	Donor	1,475	609	3.7
Salary		3,306	857	8.3
Household and stationery		736	154	1.9
Food expenses		1,249	190	3.1
Laundry expenses		291	43	0.7
**Total**		**39,657**	**10,274**	**100**

Note: calculated at the historical exchange rate over the period; sd - standard deviation

Patients were selected for treatment according to liver LT guidelines, and treatment outcomes are shown for 143 cases with MELD scores of 1.5–38 and CTP-A, B, and C scores over a 10-year period. Based on the MELD score classification, treatment costs for patients with MELD ≤ 14 points were $39,205 ± 10,786, for patients with MELD 15–20 points costs were $40,296 ± 1,517, for patients with MELD 21–27 points costs were $39,352 ± 8,718, and for patients with MELD ≤ 28 points costs were $39,812 ± 9,954, with no statistically significant difference (*p* = 0.953).

By contrast, when calculated according to the CTP score classification, the cost of treatment for CTP-A patients is $35,970 ± 6,879, for CTP-B patients $41,951 ± 12,195, and for CTP-C patients $37,396 ± 6,701, which is statistically significant (*p* = 0.015). The cost of treatment with reoperation was $44,446 ± 8,075, and the cost of treatment without reoperation was $39,589 ± 10,308, with no statistically significant difference (*p* = 0.509). (Table 4)

**Table 4:** Cost estimation of liver transplant based on the MELD score, CTP classification, and retransplantation status

	Cost /US dollar/		*P* value
	Mean	SD	
**Meld score group**			0.953†
≤ 14	39,205	10,786	
15–20	40,296	10,517	
21–27	39,352	8,718	
≥ 28	39,812	9,954	
**CTP score**			**0.015†**
CTP-A	35,970	6,879	
CTP-B	41,951	12,195	
CTP-C	37,396	6,701	
**Retransplantation**			0.509‡
No	39,589	10,308	
Yes	44,446	8,075	

MELD - Model for End-stage Liver Disease; CTP- Child-Turcotte-Pugh; Sd - Standard deviation; † - One-way ANOVA test; ‡ - Independent sample T test

## Discussion

The average cost of an LT surgery in Mongolia is $39,589 ± 10,308, with 79.6% of this amount being direct costs and 20.4% being indirect costs. Of the direct costs, 71% accounts for drugs, medical equipment, and supplies, whereas 8.6% account for salaries. Hepatitis B immune globulin (HBIG) treatment is given in an appropriate dose depending on the patient’s physical condition on the 3rd, 5th, 7th, and 14th day of the operation. This cost is included in the total cost of the LT. However, we did not estimate the cost of LT depending on the etiology. Patients with HCC have been undergoing transplants at Mongolia’s National Cancer Center since 2018. The FCHM performed LT for non-HCC, but rare cases were undertaken. Since 2022, LT procedures for children in Mongolia have been conducted at the National Center for Maternal and Child Health, with six children underwent LT between 2011 and 2021 at the FCHM under critical pediatric healthcare conditions. The cost of LT surgery for a child was not estimated separately, which is a limitation of our study.

Studies on LT treatments in developing countries are relatively limited [20]. However, the prevalence of HDV and HBV is the highest in Mongolia, the Republic of Moldova, and African countries, with a prevalence rate of over 10% [21]. The rate of LT from living donors is 1.54 per million population (pmp) in the Republic of Moldova and 0.27 pmp in African countries. The rate of LT from deceased donors is 0.6 pmp in African countries and 1.15 pmp in Moldova. In 2022, the Republic of Korea reported the highest rate of LT from living donors at 21.6 pmp, followed by Mongolia at 21.5 pmp and Türkiye at 17.3 pmp. The rate of deceased donor LT is the highest in the US at 26.6 pmp, followed by Spain at 24.1 pmp and Italy at 24 pmp [22].

In 2021, the average length of hospital stay was 32.6 ± 9.26 days, and the average duration of surgery was 13.6 ± 1.64 h, varying based on the severity of the patient’s condition. The average length of hospital stay for LT patients was 25.1 days [23]. The study performed in China noted 18.3 days for LT patients [24]. The longer length of hospital stay in Mongolia may be attributed to the practice of discharging patients only after all clinical indicators have returned to normal values post-treatment.

Compared with the results of two previous studies [13, 25], our study results are different. A significant proportion of patients in our study had a MELD score below 14, representing 43% of the total patient population. This may be explained by the system of treating patients with relatively low MELD scores in the early years of LT. With improvements in team capabilities, recent years have shown an increase in the number of surgeries performed based on high MELD scores; however, the main criteria for selecting a patient are based on the waiting list.

According to the CTP classification, the cost of LT in patients with severe liver disease and the cost calculated by the MELD score were different. In a study focused on the cost analysis of LT in Türkiye [26], the cost of treatment was not found to be related to the MELD score, whereas other studies showed a direct relationship between them [25, 27]. The estimated treatment costs, based on the MELD scores, varied but were not statistically different. Treatment costs that were estimated according to the CTP scores were higher in CTP-B patients and were statistically significant.

In this study, the average cost of LT treatment in Mongolia was estimated to be $39,589±$10,308. This result was compared to the corresponding costs reported in Argentina ($33,400) [25], Türkiye ($27,500) [26], Brazil ($17,300) [13], and the US ($40,600) [28]. The cost of treatment in Mongolia is higher than the average cost reported in other countries, and this is attributable to the dependence on imported medications, medical supplies, and reagents.

In this study, a total of six cases of children aged between 5 and 15 years who had undergone LT were examined. The cost of LT treatment in children was not calculated separately in the six cases (4.1%). Detailed cost calculations are needed for further research. The estimated pediatric LT costs in the US range from $15,700 to $26,900 [29].

A study examining the economic impact of the MELD score on liver transplant centers revealed a direct correlation between the cost of liver transplant treatment and C virus infection [30]. Similarly, a study focused on examining the cost analysis of LT in Türkiye identified the B virus infection as a direct factor contributing to the above costs [26]. Therefore, future analysis should assess if the costs of LT treatment differ according to the conditions caused by infection with B, C, and D viruses.

Based on these results, the costs for complicated cases are dependent on various factors, including any underlying liver disease, viral infections, severity of the disease, and clinical progression of the disease.

According to Mongolia’s National Health Insurance Council resolution, $30,857 is allocated to hospitals for transplant from the health insurance fund and $1,429 from the patient’s copayment [31]. According to the National Statistics Committee, the average monthly salary is $428, and the amount paid by patients is three times higher than the average salary [32]. Due to funding constraints, the Health Insurance Fund covers medical costs for up to 50 cases per year, based on hospital capacity.

The importance of this study is that LT costs were calculated based on 10-year records. Therefore, the findings could be compared with the costs of LT surgeries in other countries. As Mongolia is the 46th country in the world that has introduced LT surgery, information on treatment costs can be used as a resource for researchers in other countries.

This study did not calculate the treatment cost separately for children and adults and also based on etiology of LT treatment. Furthermore, the total cost of medicines and medical supplies was assessed based on patient records without separating the cost of medication and medical equipment. Mongolia introduced an electronic medical record system in 2016. For this reason, patients’ medical records between 2011 and 2016 were documented in paper format, delaying the research process and increasing the time taken to provide accurate data.

## Conclusion

On average, the cost of LT treatment in Mongolia is $39,589. This high-cost surgery is 8.6 times higher than the country’s gross domestic product per capita (GDP, US$4,561) [33]. Despite the capacity of medical facilities to cover treatment costs for up to 50 patients per year, there are over 300 people on the waiting list. Since 2020, with the amended Donor law, the ability to transplant from deceased donors has increased the number of surgeries performed, but the number of donors remains limited.

## Data Availability

No datasets were generated or analysed during the current study.
